# Relationship between *UGT1A9* gene polymorphisms, efficacy, and safety of propofol in induced abortions amongst Chinese population: a population-based study

**DOI:** 10.1042/BSR20170722

**Published:** 2017-10-24

**Authors:** Ying-Bin Wang, Rong-Zhi Zhang, Sheng-Hui Huang, Shu-Bao Wang, Jian-Qin Xie

**Affiliations:** Department of Anesthesiology, Lanzhou University Second Hospital, Lanzhou 730030, P.R. China

**Keywords:** Efficacy, Gene polymorphism, Induced abortion, Propofol, UGT1A9

## Abstract

The present study aimed to investigate the influence of *UGT1A9* gene polymorphisms on the efficacy of propofol in patients undergoing the painless induced abortion method. A total of 156 women seeking voluntary pregnancy termination procedures were selected for the study, and subsequently underwent painless induced abortions, following anesthesia by means of propofol administration. PCR-restriction fragment length polymorphism (PCR-RFLP) was performed to detect the polymorphisms of *UGT1A9* gene at –440C/T, –1818C/T, and –1887T/G loci. The time, effect-site concentration, and bispectral index (BIS) for the Observer’s Assessment of Alertness/Sedation (OAA/S) (up to 4 points) were observed and recorded in patients following discontinuation of propofol. The time and effect-site concentration for BIS reaching 80 in patients following the discontinuation of propofol were observed and recorded. Postoperative observations of adverse reactions, such as nausea, vomiting, and respiratory depression were all made record of. In comparison with patients with *UGT1A9* –440C/T CT and TT, those with *UGT1A9* –440C/T CC displayed shorter durations of OAA/S by up to 4 points, shorter BIS times reaching 80, as well as higher corresponding effect-site concentrations. No significant differences were detected in the patients with –440C/T, –1818T/C, and –1887T/G in incidence of nausea, vomiting, and respiratory depression. The findings of the study highlighted correlation between *UGT1A9* –440C/T gene polymorphisms and positive propofol efficacy in patients undergoing painless induced pregnancy termination procedures.

## Introduction

Induced abortion represents the most frequent outpatient surgical procedure amongst the female population. Approximately 46 million induced abortion procedures are performed worldwide on an annual basis. Reported estimations have revealed that up to 90% of abortions are performed within the first 3 months prior to the 13th week of gestation [1]. China, representing the world’s largest population, has in recent times strictly adhered to its family planning policy that has been in place since the 1970s, thus creating a scenario where advanced, safer, and more appropriate anesthesia regimens for painless induced abortions were strongly required for public health [2]. Continuous administration of parenteral analgesia during the process of induced abortion can aid in satisfactory pain relief. Propofol, as an anesthetic, plays a pivotal role in various surgical procedures and is a popular choice of anesthesia during induced abortion procedures [3]. Propofol is widely used due to its numerous advantages, including easy regulation of the anesthesia depth, short time to recover consciousness, and also less postoperative nausea and vomiting [4]. However, the pharmacokinetics of propofol is easily affected by genetic polymorphisms of metabolic enzymes [4].

The UDP-glucuronosyltransferase (UGT) family catalyzes glucuronidation of various xenobiotics and endobiotics from different species and is divided into UGT1 and UGT2 subfamilies [5]. UGT1A9 is responsible for glucuronidation of mycophenolic acid (MPA) to its inactive 7-*O*-glucuronide form and is strikingly active in the liver, kidney, and intestine [6]. In addition to the treatment of the liver, colon, kidney, testis, and ovary, UGT1A9 participates in the eliminatory process of important drugs, including irinotecan, flavopiridol, and anesthetic of propofol [7]. Reports have indicated that *UGT1A9* genotypes may act as response and toxicity predictors in colorectal cancer (CRC) patients undergoing treatment with capecitabine plus irinotecan. Particularly in patients with genotypes conferring the *UGT1A9* (dT) 9/9 genotype, who may exhibit greater antitumor responses with little toxicity [8]. The presence of UGT alleles are concerned with reduced metabolic activity as well as increasing the possibility of MPA-related adverse reactions and UGT1A9-440C>T genotype along with UGT2B7-900A>G, which signifies a risk factor for adverse reactions in pediatric kidney transplant recipients [9]. A research study conducted on 40 adult kidney transplant recipients suggested that the *UGT1A9*-440C>T genotype in the promoter region of the *UGT1A9* gene has a significant effect on MPA pharmacokinetics [10]. *UGT1A9*-440C>T polymorphisms can be important predictors of interindividual variability in MPA exposure in the pediatric population [11]. Likewise, UGT1A9 also has a rare coding single nucleotide polymorphism (SNP) T>C at 98 (UGT1A9*3) leading to a distinct decrease in glucuronidation as well as increase in MPA exposure [12]. At present, few studies regarding the polymorphisms of *UGT1A9* –1818C/T and *UGT1A9* –1887T/G have been conducted. Thus, the objective of the study was to elucidate the influence of *UGT1A9* gene polymorphisms at –440C/T, –1818C/T, and -1887T/G on the efficacy of propofol in patients undergoing painless induced abortion procedures.

## Materials and methods

### Ethics statement

This research study was conducted with the approval of the Ethics Committee of Lanzhou University Second Hospital. All patients participating in the study were provided with informed consent documentation, of which they all subsequently signed. The study protocols strictly adhered to preadvised ethical principles and all experimental procedures conducted during the present study were performed in accordance with the Declaration of Helsinki [13].

### Study subjects

Between January 2015 and January 2016, a total of 156 women (pregnancy duration: 35–60 days; aged: 29.07 ± 4.28 years; weighing: 55.45 ± 3.35 kg; height: 161.22 ± 5.88 cm; body mass index (BMI): 21.42 ± 2.00 kg/m^2^) seeking voluntary termination of pregnancy, were selected for the study. The patients subsequently underwent painless induced abortion after anesthesia with propofol. All participating females belonged to ASA-Class I and ASA-Class II [14], with 69 cases of ASA-Class I and 87 of ASA-Class II. There were 29 cases with a history of smoking, while 44 cases had a history of alcohol consumption. Patients using preanesthetic drugs, allergic to propofol and analgesics, a medical history of sedative drug addiction, bronchial asthma, and nonsteroidal anti-inflammatory drug allergy, liver and renal dysfunction or a history of peptic ulcers were excluded from the study. The patients in co-operation with medical staff were scored by means of questionnaires, 30 min prior to undergoing their respective procedures using the Hamilton anxiety scale (HAMA) method. Patients with scores of 14 of greater were considered as patients with preoperative anxiety.

### SNP detection

All patients underwent a mandatory fasting period for 10–12 h prior to morning detection. Peripheral venous blood samples (10 ml) were obtained from all the patients, added with EDTA as an anticoagulant and stored at –80°C until genomic DNA extraction. Anticoagulant blood samples were taken out and genomic DNA was extracted using a genomic DNA extraction kit (Tiangen Biotech Co., Ltd., Beijing, China). The content of genomic DNA was measured using an ultraviolet spectrophotometer. The polymorphisms of gene *UGT1A9* at –440C/T, –1818C/T, and –1887T/G were detected by PCR-restriction fragment length polymorphism (PCR-RFLP) method. Using Primer Premier 5.0 software, PCR amplification primers of –440C/T, –1818C/T, and –1887T/G in the *UGT1A9* gene were designed, examined, and synthesized by Shanghai Sangon Biological Engineering Technology Co., Ltd. (Shanghai, China) (Table 1). The –440C/T PCR reaction conditions included 3 min of predenaturation at 95°C, 36 cycles of 30 s at 94°C, 30 s at 52°C, 30 s at 70°C, and a final extension for 2 min at 70°C. The –1818C/T PCR reaction conditions included 30 s of predenaturation at 96°C, 60°C annealing for 1 min, 72°C extension for 45 s, which were cycled 11 times, with each cycle reduced by 0.5°C. The –1887T/G PCR reaction conditions included 30 s of predenaturation at 96°C, 35 cycles of 60°C annealing for 1 min, 55°C annealing for 45 s, 72°C extension for 45 s, and a final extension for 6 min at 72°C. Next, 3% agarose gel electrophoresis (100 V, 15 min) was added to the amplified PCR products (10 μl) followed by staining with Ethidium Bromide. The PCR amplification productions were analyzed by means of agarose gel electrophoresis utilizing a gel imaging system. PCR-amplified products in particular were digested for 16 h in a 37°C ice bath with specific restriction enzymes. The reaction system comprised 6 μl PCR-amplified products, 1.5 μl of 10× reaction buffer, and FspI (Toyobo Co., Ltd., Japan) (4 U), followed by the addition of sterile double distilled water which brought about the total volume to 15 μl. A 3% polyacrylamide gel electrophoresis was used to separate the enzyme-digested products, followed by Ethidium Bromide staining and analysis using the gel imaging system. The positive control was set in order to ensure the accuracy of the enzyme digestion. PCR products were identified using specific restriction enzymes HincII, XspI, and AseI (TaKaRa, Dalian, China). The sequencing, identification, and evaluation of the genotype of the corresponding locus was performed using an ABI370 type DNA sequencing instrument (ABI Company, Oyster Bay, NY, U.S.A.).

**Table 1:** The primer sequences of *UGT1A9* gene –440C/T, –1818C/T, and –1887T/G

SNP	Sequence
*UGT1A9* -440C/T	F: 5′-CACTGCGTGCGATGTATCTTAGGAA-3′
	R: 5′-TTTAGGCAACCAAATACTCTCTGTC-3′
*UGT1A9* -1818C/T	F: 5′-TTTTCAATTGTTCATTGCTA-3′
	R: 5′-CTACTCAATGGAGGACAATC-3′
*UGT1A9* -1887T/G	F: 5′-TATATGCCCGCCCCAGAG-3′
	R: 5′-TATGTCCAGCCCAATACTAGATTTT-3′

### Anesthesia and operation

Before the operation, all the 156 patients were required to adhere to a conventional fasting period. The patients were placed in the lithotomy position and a nasal catheter was used for oxygen inhalation. First, patients were given the target controlled infusion (TCI) of propofol (2 mg/kg) and then they received induced abortion after stopping eyelash reflex. The depth of anesthesia was adjudged using the bispectral index (BIS) [15]. The time of painless induced abortion surgery alone was between 10 and 30 min. The patients were injected appropriately with 25–50 mg of propofol each time if they exhibited any motions of arm twisting resistance during the procedure, which would adversely affect the operation. The final dosage of propofol administered to each patient was subsequently made record of.

### Postoperative observation

The patient gene detection results were recorded. The time, effect-site concentration, and BIS were observed and recorded when the Observer’s Assessment of Alertness/Sedation (OAA/S) score reached 4 points in patients following the discontinuation of propofol via injection. Furthermore, observations were made regarding the time and effect-site concentration and subsequently recorded when the BIS reached 80. The standard of OAA/S score amongst patients was as follows [16]: no response to squeezing the trapezius muscle (0 point); no response to slight shaking (1 point); reaction to slightly pushing head or shoulders (2 points); eyes opened following patient’s name was repeatedly called out in a loud manner (3 points); indifferent response to calling patient’s name in a normal tone (4 points); quick response observed to calling patient’s name at a normal tone (5 points). The induction time of each group was contrastingly observed, specifically including the time from the initial administration of propofol until the disappearance of the eyelash reflex, time to recover, and dosage of propofol. Visual analog scale (VAS) scores were contrastingly observed. The specific method of VAS scoring was as follows: a 10-cm line was drawn on paper. One end of the line was marked with 0 standing for no pains, and the other end of the line was marked with 10 standing for severe pain, while the middle part represented different degrees of pain on a continuum basis. Patients were required to draw marks on the line that best represented their feelings regarding the degree of pain felt. The postoperative adverse reactions, including nausea, vomiting, and respiratory depression were recorded.

### Statistical analysis

SPSS 17.0 integrated software (SPSS Inc., Chicago, IL, U.S.A.) was used for data analysis. Measurement data were exhibited as mean ± S.D. One-way factor ANOVA and least significant difference (LSD) tests were adopted for comparison amongst multiple groups. The characteristic of group representation was determined using the Hardy–Weinberg equilibrium test. A chi-square test and/or Fisher’s exact test were used in order to compare the incidence of adverse reactions amongst multiple groups. *P*<0.05 was considered as being statistically significant.

## Results

### *UGT1A9* genotypes and alleles frequency distribution

The genotypes and allele frequency distributions of the *UGT1A9* gene –440C/T, –1818C/T, and –1887T/G were consistent with the Hardy–Weinberg equilibrium in each group (all *P*>0.05), which was indicated by the characteristics of the group representation. There were 37 cases of the CC genotype of –440C/T, 88 CT genotype cases of –440C/T, 31 cases with TT genotype of –440C/T. There were 50 cases with the TT genotype of –1818C/T, 81 cases with the TC genotype of –1818C/T, and 25 cases with CC genotype of –1818C/T. There were 127 cases with TT genotype of –1887T/G and 29 cases with TG genotype of –1887T/G (Table 2).

**Table 2:** Distribution of genotype and allele frequency of *UGT1A9* gene –440C/T, –1818C/T, and –1887T/G

Genotype	Practical sampling frequency (%)	Theoretical frequency (%)	χ^2^	*P*-value
–440C/T				
CC	37 (23.72)	42 (26.96)	2.631	0.103
CT	88 (56.41)	78 (49.93)		
TT	31 (19.87)	36 (23.11)		
–1818T/C				
TT	50 (32.05)	53 (33.65)	0.681	0.410
TC	81 (51.92)	76 (48.72)		
CC	25 (16.03)	28 (17.63)		
–1887T/G				
TT	127 (81.41)	128 (82.27)	1.642	0.203
TG	29 (18.59)	26 (16.86)		

### Comparisons of baseline characteristics of patients with different *UGT1A9* genotypes

Complete clinical data of 100 patients that had fully complied with the inclusion criteria were collected. As displayed in Table 3, no significant differences were detected in patients with the *UGT1A9* gene –440C/T, –1818T/C, and –1887T/G in relation to preoperative anxiety, age, weight, height, BMI, smoking, drinking, and the duration of anesthesia (all *P*>0.05).

**Table 3:** Comparisons of baseline characteristics of patients with different genotypes in *UGT1A9* gene –440C/T, –1818C/T, and –1887T/G

Genotype	–440C/T			*P*	–1818T/C			*P*	–1887T/G		*P*
	CC (*n*=37)	CT (*n*=88)	TT (*n*=31)		TT (*n*=25)	TC (*n*=81)	CC (*n*=50)		T/T (*n*=129)	T/G (*n*=27)	
Preoperative anxiety (*n*)	27	64	22	0.978	18	59	36	0.976	94	19	0.814
Age (year)	29.65 ± 4.74	29.01 ± 3.76	28.45 ± 4.88	0.0801	28.55 ± 4.54	28.95 ± 3.91	30.48 ± 4.80	0.378	29.35 ± 3.99	27.79 ± 5.27	0.085
Weight (kg)	56.09 ± 3.79	54.80 ± 3.13	56.063 ± 0.34	0.295	55.81 ± 3.15	54.86 ± 3.24	55.35 ± 3.36	0.158	54.97 ± 3.25	56.47 ± 2.85	0.080
Height (cm)	160.62 ± 6.29	162.02 ± 5.74	160.37 ± 5.84	0.320	160.27 ± 5.90	162.17 ± 5.66	159.96 ± 6.23	0.414	161.47 ± 5.80	160.08 ± 6.11	0.244
BMI (kg/m^2^)	21.83 ± 2.18	20.25 ± 4.94	22.05 ± 3.60	0.571	22.07 ± 3.36	20.06 ± 5.12	21.85 ± 2.72	0.203	20.85 ± 4.53	21.56 ± 3.57	0.244
Smoking (*n*)	7	16	6	0.696	7	15	7	0.280	25	4	0.641
Drinking (*n*)	11	25	8	0.359	8	26	10	0.225	38	6	0.510
Anesthesia duration (min)	105.51 ± 9.27	106.74 ± 6.75	105.38 ± 8.56	0.591	105.56 ± 7.93	106.71 ± 8.10	105.19 ± 6.98	0.671	106.29 ± 7.73	105.24 ± 8.40	0.528

### Comparisons of sedative effect in patients with different *UGITA9* genotypes

Following the discontinuation of TCI of propofol, in comparison with the patients with the *UGT1A9* –440C/T CT and TT, those with the *UGT1A9* –440C/T CC exhibited a shorter duration of OAA/S score totaling 4 points, a shorter time of BIS reaching 80, and higher effect-site concentration (all *P*<0.05). No significant differences regarding the indexes of patients with *UGT1A9* –1818T/C and –1887T/G were detected (all *P*>0.05) (Table 4).

**Table 4:** Comparisons of sedative effect in patients with different *UGITA9* genotypes

Item	–440C/T			*P*	–1818T/C			*P*	–1887T/G		*P*
	CC (*n*=37)	CT (*n*=88)	TT (*n*=31)		TT (*n*=25)	TC (*n*=81)	CC (*n*=50)		T/T (*n*=129)	T/G (*n*=27)	
OAA/S up to 4 points											
Time (min)	5.00 ± 1.24*	5.52 ± 0.90	5.70 ± 0.93	0.008	5.27 ± 1.13	5.45 ± 0.74	5.71 ± 0.93	0.090	5.39 ± 1.06	5.69 ± 0.77	0.165
BIS	70.67 ± 7.35	71.24 ± 8.05	69.39 ± 9.42	0.032	71.55 ± 7.87	70.42 ± 8.10	69.36 ± 8.92	0.160	70.90 ± 8.05	69.38 ± 9.05	0.384
Effect-site concentration (μg.ml^−1^)	3.67 ± 0.96*	3.21 ± 0.78	3.20 ± 0.69	0.011	3.39 ± 0.95	3.28 ± 0.64	3.20 ± 0.69	0.549	3.34 ± 0.85	3.18 ± 0.67	0.359
BIS reaching 80											
Time (min)	7.91 ± 2.60*	8.92 ± 1.67	9.10 ± 1.99	0.019	8.58 ± 2.22	8.74 ± 1.70	8.99 ± 1.94	0.624	8.64 ± 2.01	9.12 ± 2.18	0.268
Effect-site concentration (μg.ml^−1^)	2.08 ± 0.41	2.02 ± 0.29	1.97 ± 0.36	0.041	2.05 ± 0.36	2.00 ± 0.26	1.98 ± 0.34	0.496	2.03 ± 0.33	1.96 ± 0.38	0.331

*, compared with the CT and TT groups, *P*<0.05.

### Comparisons of time to disappearance of eyelash reflex, recovery, dosage of propofol, and VAS score of patients with different *UGITA9* genotypes

The time for disappearance of eyelash reflex and recovery was significantly shorter in patients with –440C/T CC than those with –440C/T CT and TT (both *P*<0.05). The dosage of propofol as well as the VAS score of patients with –440C/T CC were significantly lower than those with –440C/T CT and TT (both *P*<0.05). The time for disappearance of eyelash reflex and recovery, dosage of propofol, and VAS score displayed no significant difference in patients with –1818T/C and –1887T/G (all *P*>0.05) (Table 5).

**Table 5:** Comparisons of time for disappearance of eyelash reflex and recovery, dosage of propofol, and VAS score of patients with different UGITA9 genotypes

Genotype	–440C/T			*P*	–1818T/C			*P*	–1887T/G		*P*
	CC (*n*=37)	CT (*n*=88)	TT (*n*=31)		TT (*n*=25)	TC (*n*=81)	CC (*n*=50)		T/T (*n*=129)	T/G (*n*=27)	
Time for disappearance of eyelash reflex (s)	53.93 ± 12.13*	58.86 ± 9.11	60.43 ± 7.04	0.010	59.61 ± 7.22	56.66 ± 11.26	59.66 ± 7.74	0.160	57.60 ± 10.02	60.40 ± 7.68	0.178
Time for recovery (min)	4.91 ± 1.35*	6.37 ± 1.06	6.08 ± 1.13	<0.0001	6.36 ± 0.99	5.75 ± 1.46	6.00 ± 0.99	0.097	5.91 ± 1.32	6.12 ± 1.10	0.441
Dosage of propofol (mg/kg)	2.61 ± 0.76*	3.28 ± 0.64	3.11 ± 0.71	<0.0001	3.28 ± 0.65	2.93 ± 0.73	3.23 ± 0.75	0.097	3.08 ± 0.75	3.03 ± 0.68	0.749
VAS score	1.52 ± 0.43*	2.09 ± 0.42	1.94 ± 0.35	<0.0001	2.05 ± 0.45	1.87 ± 0.53	1.92 ± 0.34	0.240	1.92 ± 0.48	1.91 ± 0.36	0.918

*, compared with the CT and TT groups, *P*<0.05.

### Comparisons of postoperative adverse reactions in patients with different *UGITA9* genotypes

Nausea and vomiting were the most common adverse reactions recorded, post-propofol administration. However, the incidence of nausea and vomiting was very low in addition to the fact that no significant difference in relation to the incidence of postoperative nausea and vomiting amongst patients with –440C/T, –1818T/C, and –1887T/G were observed (all *P*>0.05). No obvious respiratory depression occurred amongst patients who participated in the study (Table 6).

**Table 6:** Comparisons of postoperative adverse reactions in patients with different UGITA9 genotypes

Genotype	*n*	Mild nausea	Moderate nausea	Severe nausea	Vomiting	Respiratory depression
–440C/T						
CC	37	8	4	1	2	0
CT	88	24	20	6	10	1
TT	31	8	5	2	3	0
–1818T/C						
TT	25	7	3	1	2	0
TC	81	25	17	6	8	1
CC	50	8	9	2	5	0
–1887T/G						
T/T	129	36	27	8	12	1
T/G	27	4	2	1	3	0

## Discussion

A deep sense of fear and pain are not uncommon in the process of clinical check-up and treatment surgery. As a result of this, the use of anesthetics to generate a transient loss of consciousness is a urgent clinical requirement. However, anesthesia is not without its own set of risk factors as well as the requirement of a diligent process of anesthesia preparation, including anesthesia equipment, clinical skills of anesthesiologists, selection of anesthetics, and dose deviation of anesthetic drugs, which without adequate attention may result in loss of life. The role of genetic factors is progressively being paid more attention due to the clinical consensus that they play a critical role amongst many factors affecting an individual’s variation in regard to the effect of anesthesia [17]. Gene variation may take place at the genetic level, and SNP remains the most stable as well as the most common genetic variation at present identified amongst individuals [18]. SNPs are abundant and responsible for majority of the observed phenotypic variations in plants. Likewise, individual SNPs may directly contribute to phenotypic variations [18,19]. However, not all the variants will result in clinical implications. Thus, it is important to assess the effect of genetic variants on an individual level in regard to varying drug efficacies. The present study aimed to evaluate the association between the *UGT1A9* gene polymorphisms and the resultant effects of propofol during induced abortion procedures, in order to provide a theoretical basis for more reasonable and safer dosage regimens of for clinical propofol use.

A key finding of the study revealed that following the discontinuation of TCI of propofol, when compared with *UGT1A9*-440C/T CT and TT patients, those with *UGT1A9*-440C/T CC exhibited shorter durations of OAA/S with values up to 4 points, shorter times of BIS reaching 80, as well as greater corresponding effect-site concentrations. BIS is a type of detection method clinically employed to measure the depth of sedation and acts as a guide for the management of anesthesia. At present, BIS is considered to be a good indicator with its advantages including a greater accuracy while monitoring the recovery of consciousness in patients after procedures utilizing general anesthesia. Thus, it results in simpler extubation while monitoring the state of consciousness of a patient [20]. The application of BIS in a study of monitoring the depth of anesthesia has attracted great attention [21]. Studies show that BIS can reflect the sedation of a variety of anesthetic drugs [22]. BIS can well reflect the depth of sedation of anesthetic drugs and is an accurate and sensitive index to evaluate the effect of anesthesia [23]. A study of 140 cardiac surgery patients under propofol during their postoperative phase reported that although it does not reflect the physiological structures of movement suppression, allows an even more accurate prediction due to its ability to closely correlate with the dose of propofol administered [24]. The BIS index is positively correlated with OAA/S scale. Additionally, lower the OAA/S value, deeper the level of sedation is [25,26]. As a result, the depth of sedation of patients with *UGT1A9*-440C/T CC administered with propofol, is deeper than that of patients with *UGT1A9*-440C/T CT and TT according to the OAA/S value.

Another significant finding of our study, was in relation to the comparison made between the loss time of eyelash reflex, recovery time, dosage of propofol, and VAS score of patients with *UGT1A9*-440C/T CT and TT, those with *UGT1A9*-440C/T CC showed shorter loss time of eyelash reflex and recovery time, and less dosage of propofol and a lower VAS score. The present results indicated that the effect of propofol was related to the *UGT1A9* gene polymorphism during the induced abortion procedure, which also suggested that the anesthetic effect in patients with *UGT1A9*-440C/T CC was better than in patients with *UGT1A9*-440C/T CT and TT. UGT1A9 also has a rare coding SNP T>C at 98 (UGT1A9*3), which resulted in a dramatically decreased level of glucuronidation [11]. SNP is the most important genetic factor responsible for individual differences in drug metabolism and efficacy. The polymorphism of gene sequences is one of the significant reasons for the different responses observed amongst different individuals who receive the same drug [27]. UGT1A9 conjugates a great amount of endogenous and exogenous substrates, including estrogens and thyroid hormones, acetaminophen, SN-38 (an active metabolite of irinotecan), and phenols [28,29]. Individual differences exist in relation to the expression of UGT1A9 and the activity of glucose. *UGT1A9*-440C/T is a polymorphic locus with a particularly high mutation rate and may affect its phenotype, *UGT1A9*. Abundant interindividual variability in MPA exposure is illustrated in part with the presence of the *UGT1A9-*440C>T/-331T>C genotype in the promoter region of the *UGT1A9* gene (*P*=0.005), which was demonstrated in a study with 40 adult kidney transplant recipients [11]. The *UGT1A9*-440C/T distribution in the intron region can increase the enzyme content and activity of glucose of UGT1A9, and when compared with *UGT1A9*-440C/T CT and TT, *UGT1A9*-440C/T CC exhibits a distinctly higher SN-38 glucose activity rate. Moreover, it has been reported in a previous study that sex difference exists in the roles of UGT1A9 in its metabolism that propofol participates [30]. There have also been correlations made between sex and UGT1A9 polymorphisms in regard to propofol and results reported in a previous pilot study, which indicated that in comparison with males, more rapid propofol metabolism could occur in female.

In conclusion, the findings of the present study have highlighted the significant role played by *UGT1A9* gene polymorphisms in painless induced abortion procedures linked to the effects of propofol. The dosage of propofol can be adjusted according to the individual patient in the event of the possession of different genotypes, which may enhance the effects of anesthesia, reduce the dose of drugs administered, as well as the postoperative adverse reactions experienced. However, the number of the study subjects is limited and it must be noted that our findings need to be further validated by additional functional studies and well-designed larger molecular epidemiological studies that involve diverse ethnic populations.

## Abbreviations

BIS: bispectral index
BMI: body mass index
MPA: mycophenolic acid
OAA/S: Observer’s Assessment of Alertness/Sedation
SNP: single nucleotide polymorphism
TCI: target controlled infusion
UGT: UDP-glucuronosyltransferase
VAS: visual analog scale
